# Precision therapy for three Chinese families with maturity-onset diabetes of the young (MODY12)

**DOI:** 10.3389/fendo.2022.858096

**Published:** 2022-08-03

**Authors:** Juyi Li, Xiufang Wang, Huihui Mao, Li Wen, Aiping Deng, Yarong Li, Hongmei Zhang, Chao Liu

**Affiliations:** ^1^ Department of Pharmacy, The Central Hospital of Wuhan, Tongji Medical College, Huazhong University of Science and Technology, Wuhan, China; ^2^ Department of Pain, The Central Hospital of Wuhan, Tongji Medical College, Huazhong University of Science and Technology, Wuhan, China; ^3^ Department of Nephrology, The Central Hospital of Wuhan, Tongji Medical College, Huazhong University of Science and Technology, Wuhan, China; ^4^ Department of Traditional Chinese Medicine and Ethnic Medicine, Guangxi Institute for Food and Drug Control, Nanning, China; ^5^ Department of Endocrinology, The Central Hospital of Wuhan, Tongji Medical College, Huazhong University of Science and Technology, Wuhan, China; ^6^ Hubei Key Laboratory of Diabetes and Angiopathy, Hubei University of Science and Technology, Xianning, China

**Keywords:** *ABCC8*, MODY12, precision therapy, mutation, whole exome sequencing

## Abstract

Maturity-onset diabetes of the young (MODY) is rare monogenic diabetes. However, MODY is often undiagnosed or misdiagnosed. In this study, we aimed to investigate the pathogenic gene for diabetes and provide precise treatment for diabetes patients in three families. Three families with suspected MODY were enrolled and screened for germline mutations using Whole exome sequencing (WES). Candidate pathogenic variants were validated in other family members and non-related healthy controls. Three heterozygous missense mutations in the *ABCC8* gene (NM_001287174), c.1555 C>T (p.R519C), c.3706 A>G (p.I1236V), and c.2885 C>T (p.S962L) were found in families A, B, and C, respectively. All mutation sites cosegregated with diabetes, were predicted to be harmful by bioinformatics and were not found in non-related healthy controls. Two probands (onset ages, 8 and 12 years) were sensitive to glimepiride. However, an insufficient dose (2 mg/day) led to ketoacidosis. When the dosage of glimepiride was increased to 4 mg/day, blood sugar remained under control. A dose of 4 mg glimepiride daily also effectively controlled blood sugar in an adult patient 25-year-old. In addition, all patients were sensitive to liraglutide, which could control blood sugar better. These data suggest that *ABCC8* was the pathogenic gene in three families with diabetes. Glimepiride (2 mg/day) was not effective in controlling blood sugar in children with *ABCC8* mutations, however, 4 mg/daily glimepiride was effective in both adults and children. Moreover, liraglutide was effective in controlling blood sugar in both adults and children with *ABCC8* mutations.

## Introduction

Maturity-onset diabetes of the young (MODY) is an autosomal dominant mode of inheritance and a monogenic form of diabetes, characterized by an early age at onset (usually at ≤25 years of age) and impaired insulin secretion with minimal or no defects in insulin action (in the absence of coexisting obesity) (1, 2). To date, 14 genes have been identified to be responsible for MODY: *HNF4A* (MODY1), *GCK*(MODY2), *HNF1A* (MODY3), *PDX1* (MODY4), *HNF1B*(MODY5), *NEUROD1* (MODY6), *KLF11* (MODY7), *CEL*(MODY8), *PAX4*(MODY9), *INS* (MODY10), *BLK*(MODY11), *ABCC8* (MODY12), *KCNJ11* (MODY13), and *APPL1*(MODY14) (2–4). MODY3 and MODY2 accounted for 80 – 90% of MODY in Caucasians (5, 6). MODY3 accounted for 30– 50% of the MODY cases, MODY1 and MODY5, respectively, accounted for 5% of the cases, and only a small proportion (< 1%) of the MODY cases were caused by other MODY subtypes (7–9). However, MODY was detected in only 9% of the patients in China with diabetes, therefore, most of the MODY genes in the Chinese population are still unknown (5).

The pathogenesis of MODY is highly genetically heterogeneous; thus, available treatment options are very different. For instance, *GCK*-MODY patients do not require treatment, whereas *HNF1A*- and *HNF4A*-MODY patients can be treated with low dose sulfonylurea and *HNF1B*-MODY patients require insulin treatment (10–13). Therefore, personalized medicine in MODY subtypes is essential. However, diagnosing MODY poses a challenge for physicians, with most cases remaining unidentified. This makes rapid diagnosis of MODY subtype of vital importance to patients and their families, because it provides a basis for individualized treatment and prognosis.

In this study, we report three heterozygous missense mutations, c.3706 A>G (p.I1236V) in family A, c.1555 C>T (p.R519C) in family B, and c.2885 C>T (p.S962L) in family C in the *ABCC8* gene (NM_001287174). Blood sugar levels were better controlled in these families after adjusting the drug treatment plan according to the patient’s genetic test results.

## Materials and methods

### Participants

This study was approved by the Ethics Committee of the Central Hospital of Wuhan (2016–2). Informed consent was obtained from the patients and their families. Researchers had access to clinical information and medical records of the participants. Patients with a clinical diagnosis of MODY were included in the study and the criteria were age at diagnosis of ≤25 years, negative islet autoantibodies, and a family history of diabetes.

### Whole-exome sequencing (WES)

Genomic DNA was extracted from the participants’ peripheral blood samples (TIANGEN DNA extraction kit, Beijing, China). We used the Agilent SureSelect Human All Exon V6 kit for capture, followed by sequencing on the *Illumina* hiSeq2500 system, reaching an average depth of approximately 50×. The reference genome for aligning WES was human GRCh37/hg19. SNPs and indels were called by SAMtools, correcting for overestimated mapping quality from Burrows-Wheeler Alignment tool (14).

### Genetic analysis

Variants were filtered to retain protein-altering variants (including non-synonymous, frame shift and splicing, and excluding non-exonic and synonymous variants), and to exclude those with minor allele frequency (<0.001) according to certain public databases (1000genomes, ExAc, dbSNP, ESP and gnomAD). The effects of the identified variants were evaluated using silico prediction tools, namely SIFT, PolyPhen2_HDIV, PolyPhen2_HVAR, LRT, Mutation Taster, Mutation Assessor and FATHMM (15). We selected genes previously implicated in monogenic diabetes, genes with important roles in the beta cell, and genes associated with type 2 diabetes or fasting glucose (16). Variants of uncertain significance (VUS), defined as functional significance, are not recorded in the public database and there are no relevant reports in the literature, such as novel missense variants, variants with conflicting evidence, or variants with insufficient evidence (17).

### Sanger sequencing

Candidate variants in the proband and his family were confirmed by direct Sanger sequencing of the PCR products. Afterwards, the candidate variants that cosegregated with diabetes were selected for further analysis. The *ABCC8* gene was amplified by PCR using forward primer (proband A) 5′-TCAAGGCCTCCTGCTTCTGT-3^′^ and reverse primer 5′-TGTCCAGGCCTCAGCTTCTT-3^′^, forward primer (proband B) 5′- AGCAGGAAGGCTTGGTGT-3^′^andreverse primer 5′- TTGGAGGAGGTGGGGATT-3^′^, forward primer (proband C) 5′- ACACCCAGAACCCCAAACCT-3^′^and reverse primer 5′- GTGTCTGTCTGCCCCTCCCT-3^′^. The PCR conditions were as follows: 95 C for 5 min; 35 cycles of 95 °C for 1 min, 60 °C for 30 sec, and 72 °Cfor 1 min; and then 72 °C for 10 min. The PCR products were directly sequenced (Beijing Genomics Institute, Wuhan, China) (18).

### Medication plan adjustments and follow-up

The patients’ treatment plans were adjusted based on genetic test results and previous literature reports. For example, patients with ABCC8 gene mutations are more sensitive to sulfonylurea drugs. Therefore, according to the results of the gene test, patients with ABCC8 mutations can use sulfonylureas for hypoglycemic treatment. Blood glucose levels were regularly monitored (every day), and the HbA_1C_ levels were controlled for two months.

## Results

### Medical history

Proband A was an eight-year-old female, who was admitted to the hospital for a stuffed nose and sleep snoring and was diagnosed with type 1 diabetes at the age of eight. Her body mass index (BMI) was14.0 kg/m^2^. The proband’s parents (III-1 and III-2) were 38 years old and non-diabetics, their BMIs were 26.1 kg/m^2^and 22.0kg/m^2^. The proband’s grandmother (II-1) was diagnosed with diabetes at the age of 51, and her BMI was 20.0kg/m^2^.The father of the proband’s grandmother (I-1) died from diabetes-related causes.

The proband B was a 12-year-old male, who was admitted to the hospital for the following symptoms: dry mouth, polydipsia, polyuria, and fatigue. The physical examination revealed fasting blood glucose of 14.28mmol/Land a BMI of 26.9 kg/m^2^. The proband’s father (III-1), mother (III-2), grandfather (II-1), maternal grandfather (II-4), and maternal grandfather’s sister (II-5) and brother (II-6) were diagnosed with diabetes at the ages of 43, 41, 59, 69, 59, and 61, respectively, and their BMIs were less than 28.0 kg/m^2^.

Proband C was a 25-year-old male, who was admitted to the hospital for dry mouth, polydipsia, polyuria, and fatigue, and was diagnosed with diabetes and ketoacidosis. His BMI was 27.8 kg/m^2^. He was prescribed insulin, which resulted in well-controlled blood sugar levels. The proband’s mother (II-4) and maternal grandfather (I-4) were diagnosed with diabetes at the ages of 41 and 60, respectively, and the proband’s mother (II-4) had a BMI of 22.8kg/m^2^.

**Figure 1:**
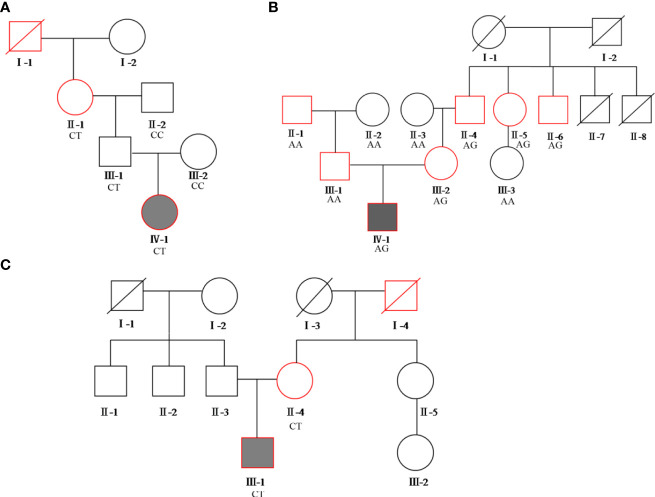
Pedigree of the three families. Squares represent males, while circles represent females. The red border represents diabetes, the blackened box represents the proband. **(A)** proband A’s family pedigree; **(B)** proband B’s family pedigree; **(C)** proband C’s family pedigree. The genotypes of each variation of c.1555 C>T, c.3706 A>G, and c.2885 C>T in the *ABCC8* gene are shown in families **(A–C)**, respectively.

The BMI of all family members of the diabetes patients was in the normal range or had not reached the obese range (> 28.0kg/m^2^). The pedigree of the three families is presented in **Figure 1**.

### Clinical characteristics

The clinical characteristics of the three probands are shown in **Table 1**. Proband A’s laboratory analysis revealed that her HbA_1C_ level was 12.8%, her fasting plasma glucose (FPG) level was 18.81 mmol/L, and her fasting c-peptide was 2.35 ng/ml. She tested positive for urine glucose (2+) and urine ketones (1+), and her triglyceride levels were high (50.21 mmol/L), whereas she tested negative for all diabetes autoantibodies. Ultrasound examination of the liver, kidney, pancreas, bladder, ureter, spleen, and gall showed that organ morphology was normal. Although she was prescribed high-dose insulin and oral hypoglycemic agents (insulin glulisine, 20 units, three times a day; degludec, 30 units/night; metformin tablets, 850 mg twice a day; pioglitazone hydrochloride and metformin hydrochloride tablets, 15 mg/500mg, twice a day, and notified her guardian) for two days, her FPG levels did not drop. However, her triglyceride levels decreased to 12.6 mmol/L. The test results of proband A’s grandmother (II-1) were as follows: HbA_1C_, 7.1%; FPG, 11.7 mmol/L; fasting c-peptide, 1.60 ng/ml, and positive (1+) urine glucose, whereas ketones in the urine and diabetes autoantibodies were all negative. She (II-1) had diabetic retinopathy and diabetic neuropathy and was previously prescribed acarbose and insulin. However, her (II-1) blood sugar was not well controlled.

**Table 1:** Physical and laboratory examination.

Parameter	Proband A	Proband B	Proband C
Age (year)	8	12	25
Gender	Female	Male	Male
BMI, kg/m^2^	14.0	26.9	27.8
Fasting urine sugar	++	4+	4+
Ketones in urine	+	Neg	3+
Fasting plasma glucose (FPG), mmol/L	18.81	14.28	13.52
Fasting C-peptide, ng/mL	2.35	1.80	1.30
2-h fasting C-peptide, ng/mL	4.56	3.10	1.70
HbA1c, %	12.8	12.1	11.2
Albumin, g/L	65.5	44.3	39.9
Globulin, g/L	59.7	24.6	30.1
Alanine aminotransferase, U/L	35.0	56.6	92.4
Aspartate aminotransferase, U/L	32.0	27.1	47.2
Creatinine, μmol/L	42.0	45.0	56.8
Uric acid, μmol/L	269	707	801
TG, mmol/L	50.21	3.89	16.78
TC, mmol/L	8.46	5.94	7.36
HDL-C, mmol/L	0.67	1.12	0.51
LDL-C, mmol/L	1.50	3.66	1.70
hsCRP, mg/L	0.10	–	0.40
Anti-GAD	Neg	Neg	Neg
Anti-IAA	Neg	Neg	Neg

The results of biochemical indicators when the three probands were admitted to the hospital.-: no detection; HbA1c, glycosylated hemoglobin; HDL-C, high density lipoprotein cholesterol; LDL-C, low density lipoprotein cholesterol; TG, triglyceride; TC, total cholesterol; hsCRP, hypersensitive C reactive protein; GAD, glutamic acid decarboxylase antibodies; IAA, insulin autoantibodies; Neg, negative. +, it means positive. The more plus signs, the greater the value.

Physical examination revealed that proband B presented with hyperlipidemia, hyperuricemia (707 μmol/L), and mild fatty liver. Additionally, HbA_1C_ was 12.1%, urine glucose was positive (4+), and fasting c-peptide was 1.8 ng/ml, while ketones in the urine and diabetic autoantibodies were all negative. There were no obvious abnormalities in other assessments. Although proband B’s islet function was normal, his fasting and two-hour postprandial blood glucose were high for a child. Hence, the doctor had been administering insulin, which led to the patient’s blood glucose being under control. Proband B’s father (III-1) was prescribed metformin and gliclazide sustained-release tablets, his HbA_1C_ levels were 7.4%, FPG was 9.87 mmol/L, and he additionally presented with diabetic nephropathy and neuropathy. Proband B’s mother (III-2) and maternal grandfather (II-4) were prescribed insulin, metformin, and linagliptin. However, they (III-2 and III-4) did not achieve optimal outcomes since their HbA_1C_ and FPG levels were approximately 10% and11 mmol/L, respectively. The maternal grandfather’s sister (II-5) and brother (II-6) of proband B were prescribed insulin and acarbose, respectively, their FPG levels reached 23 mmol/L at times, and both had diabetic retinopathy and neuropathy.

Proband C’s laboratory analysis revealed that his HbA_1C_ level was 11.2%, FPG level was 13.52 mmol/L, fasting c-peptide was 1.30 ng/ml, urine glucose tested positive (4+), as well as ketones in urine (3+), and triglyceride levels were high (16.78 mmol/L). However, diabetic autoantibodies were all negative. Ultrasound examination indicated that all organs were normal and he exhibited no diabetes-related complications. Proband C was prescribed liraglutide for three days (0.6 mg/daily). Nonetheless, FPG and triglyceride levels decreased to 9.64 mmol/L and 6.11 mmol/L, respectively. The results of proband C’s mother (II-4) were as follows: HbA_1C_ was 8.6%, the FPG level was 9.65 mmol/L, and she presented with complicated diabetic neuropathy. Although she (II-4) had been using metformin and acarbose, her blood sugar level was not under control.

### Genetic and bioinformatic analyses

**Figure 2:**
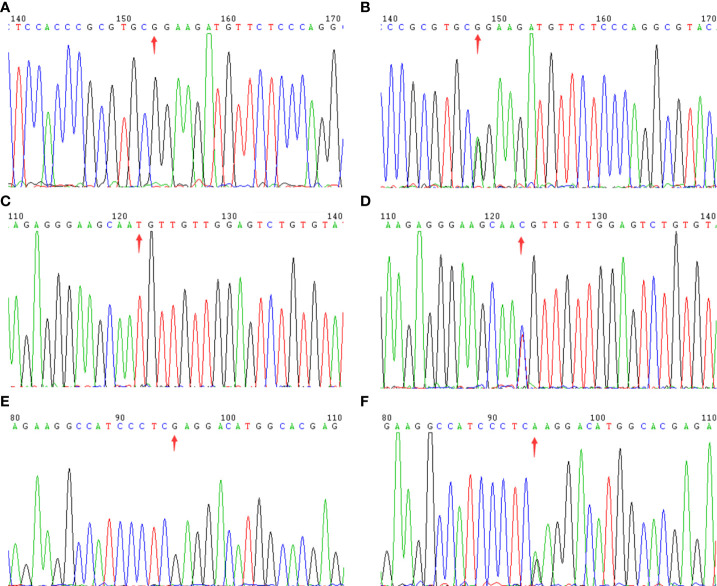
The sequencing chromatogram. Arrows indicate the changed position of the mutation in *ABCC8* gene. Family A: **(A)** (wild type) and **(B)** (mutant type); Family B: **(C)** (wild type) and **(D)** (mutant type); Family C: **(E)** (wild type) and **(F)** (mutant type).

WES details are shown in **Table 2**. WES showed that the variants of each subject ranged from 110,000 to 130,000, including SNPs and InDels in probands A, B, and C. Variants were filtered as previously described and the defined panel of MODY genes (*HNF4A*, *GCK*, *HNF1A*, *PDX1*, *HNF1B*, *NEUROD1*, *KLF11*, *CEL*, *PAX4*, *INS*, *BLK*, *ABCC8*, *KCNJ11*, and *APPL1*) were firstly evaluated (15). *ABCC8* was found to be a candidate pathogenic gene in all three families, and no other known MODY pathogenic variants were identified. The details are as follows. A known mutation was found in proband A: chromosome 11, position 17464342, c.C1555T, NM_001287174, p. R519C, namely, rs1057467571. This mutation has a very low frequency (25/251142, GnomAD_exome; 11/121224, ExAC; 1/31398, GnomAD) in the public database, but has not been reported in Chinese populations, and its clinical significance is not reported in ClinVar. Despite being a known mutation, its function remains unclear. In proband B, we found a novel mutation, chromosome 11, position 17419936, c.A3706G, NM_001287174, p. I1236V. In proband C, we discovered a known mutation, chromosome 11, position 17428939, c.C2885T, NM_001287174, p. S962L, namely, rs748931549.There is no reported frequency for this mutation in the public database. Its clinical significance is not reported in ClinVar. Despite being a known mutation, its function remains unclear. The three mutation sites were then sequenced and validated in other members of their respective families (**Figure 2**). Although we did not find them in non-related healthy controls (n=200), among the members of family A, the proband A’s father (currently 38 years old) also carried this mutation site (c.C1555T) but did not have diabetes. In family B, the mutation site (c.A3706G) was inherited from the mother (III-2), and the other patients (II-4, II-5, and II-6) all carried this mutation. Although proband B’s father may also have a genetic history of diabetes, no suspicious pathogenic site has been found so far; therefore, the pathogenic site of diabetes should come from probands B’s mother. In family C, the mutation site (c.C2885T) co-segregated with diabetes (**Supplementary Table 1**). However, all three mutation sites were predicted to be harmful mutations by multiple bioinformatics software, and the mutation sites were highly conserved in several species (**Supplementary Tables 2**, **3**). Consequently, we speculated that these three mutation sites are pathogenic mutations in these families, and that they all belong to the *ABCC8*-MODY12 type.

**Table 2:** Whole exome sequencing detail.

Exome Capture Statistics	Proband A	Proband B	Proband C
Total	100067854 (100%)	72,395,146 (100%)	73,319,066 (100%)
Duplicate	20983854 (21.03%)	11,878,602 (16.42%)	17,446,625 (23.81%)
Mapped	99777578 (99.71%)	72,360,733 (99.95%)	73,276,960 (99.94%)
Properly mapped	99291082 (99.22%)	72,006,112 (99.46%)	72,752,410 (99.23%)
PE mapped	99554292 (99.49%)	72,333,344 (99.91%)	73,241,380 (99.89%)
SE mapped	446572 (0.45%)	54,778 (0.08%)	71,160 (0.10%)
Initial_bases_on_target	60456963	60,456,963	60,456,963
Initial_bases_on_or_near_target	136297444	136,297,444	136,297,444
Total_effective_yield (Mb)	14928.63	10,826.59	10,961.10
Effective_yield_on_target (Mb)	10851.51	7,914.93	8,271.13
Fraction_of_effective_bases_on_target	72.7%	73.1%	75.5%
Fraction_of_effective_bases_on_or_near_target	94.7%	94.0%	95.1%
Average_sequencing_depth_on_target	179.49	130.92	136.81
Bases_covered_on_target	60169235	59,940,193	59,758,460
Coverage_of_target_region	99.5%	99.1%	98.8%
Fraction_of_target_covered_with_at_least_100x	64.4%	52.0%	52.2%
Fraction_of_target_covered_with_at_least_50x	86.2%	77.8%	76.4%
Fraction_of_target_covered_with_at_least_20x	96.0%	92.1%	90.8%
Fraction_of_target_covered_with_at_least_10x	98.2%	95.9%	95.0%
Fraction_of_target_covered_with_at_least_4x	99.2%	98.0%	97.5%

### Precise treatment based on the patient’s genotype

Genetic testing confirmed that the above mentioned families belong to the MODY12 subtype. This information, combined with results reported in the literature, led to recommending that these patients replace current medical treatment with sulfonylurea drugs.

The proband A was administered insulin (insulin aspart injection, 12 units, subcutaneous injection before meals, and insulin glargine injection, 12 units, subcutaneous injection before 9 pm) due to high fasting blood sugar and positive urine ketones, as he was a child. However, the proband A’s fasting blood sugar was still high after two days of treatment. Then, the insulin dose was increased to 50 units, but the fasting blood glucose still did not decrease (the fasting blood sugar levels remained between 12 and 18 mmol/L), even when acarbose and metformin enteric-coated tablets were used. The results of the genetic test suggested that proband A was classified as MODY12, and there is no evidence of sulfonylureas being used previously to treat pediatric diabetes in China. Thus, we tried to utilize the relatively safe glimepiride combined with insulin on the child. When glimepiride was added, the child’s fasting blood glucose rapidly decreased. After a week, the child’s fasting blood glucose level was in the normal range (< 6.1 mmol/L).We then slowly reduced the dose of insulin (by 5 units a week), and shortly the insulin administration ceased, and only glimepiride (2 mg/day) was used. Nonetheless, after two weeks, proband A developed diabetic ketosis, necessitating the use of glimepiride (2 mg/day) in combination with a low-dose insulin aspart injection (5 units/day, twice a day) for hypoglycemia. Then, the dosage of glimepiride (4 mg/day) was increased and insulin administration was stopped. The results from follow-up tests in the following two months showed that the child’s fasting blood sugar (< 6.1 mmol/L) and glycation levels (< 6.5%) were within the normal ranges.

Proband B, who is currently 13 years old, presented with type 2 diabetes. Therefore, we replaced the previously prescribed insulin aspart with a new type of hypoglycemic drug, liraglutide injections at 1.2 mg/day, and notified his guardian. This drug’s hypoglycemic properties produced very effective results. After two months of follow-up, the patient’s blood sugar levels were under control and within the normal range (HbA1c < 6.5%). Taking into consideration that this proband B is MODY12 and a pediatric patient, liraglutide injections yielded very positive effects in terms of blood sugar reduction. Liraglutide was then gradually reduced. When glimepiride was added (2 mg/day), until liraglutide administration ceased, the blood glucose levels remained within a controlled range. However, ketosis appeared shortly, and thus, liraglutide use was continued. Then, the dosage of glimepiride was increased (4 mg/day) and liraglutide was stopped. The patient’s blood glucose returned to the normal range (HbA1c < 6.5%).

Proband C was also very sensitive to liraglutide injections (1.2 mg/day). Thus, this hypoglycemic treatment was very effective. Since injections are more problematic, whereas oral medication is relatively simple to administer, we recommended glimepiride 4mg/day as a medication plan based on the patient’s MODY12 type. After two months of follow-up, the patient’s blood glucose returned to a reasonable level (HbA1c < 6.5%).

Currently, other adult MODY12 patients in these three families were mainly using insulin or insulin combined with metformin or acarbose, with deficient results on blood sugar control. However, according to each patient’s genotype, they were slowly switched to glimepiride (4 mg/day), and their blood glucose levels were controlled after 2-3 months and the HbA_1C_levels approached normal ranges(< 6.5%). All diabetes patients continued to be treated with glimepiride (4 mg daily).

## Discussion

We have identified three heterozygous *ABCC8* mutations in three different families. Two of these mutations are known mutations (p. R519C and p. S962L). However, their functional significance has not been reported yet. The remaining mutation was novel (p. I1236V). The three mutations (p. R519C, p. I1236V, and p. S962L) are highly likely to be pathogenic for the following reasons: their mutation sites were predicted to be harmful by bioinformatics software, the residues are highly conserved, and carriers of these mutation sites are very sensitive to glimepiride. The mutation p. R519Cwas located in the domain of ABC transmembrane type-1 1, whereas the mutation p. I1236Vwas located in the domain of ABC transmembrane type-1 2.Yet, functional studies are required to demonstrate that these mutations increase K_ATP_ channel activity or may result in loss-of-function and cause diabetes.

*The ABCC8*gene encodes the sulfonylurea receptor 1 (SUR1) subunit of the ATP-sensitive potassium (K_ATP_) channel in pancreatic beta cells, which regulate insulin secretion. Mutations in the *ABCC8* gene cause the overactivity or underactivity of the K_ATP_ channel, and thereby, a variety of phenotypes (19).Previous studies suggested an association between adult-onset diabetes and mutations in the *ABCC8* gene (20). *ABCC8* mutations can cause MODY in patients whose clinical features are similar to those with *HNF1A/4A*MODY *(* 19), and some patients with *ABCC8* mutations were able to change from insulin to oral glibenclamide therapy (21, 22), which means that patients with *ABCC8*presented with T2DM-like phenotypes. Exome sequencing identified many variants, most of which lacked the corresponding functional annotations and had no functional significance. However, a few variants were functionally significant. *ABCC8* variants are associated with T2DM. Therefore, exome sequencing studies identified rare variants with functional significance in *ABCC8*, which were enriched in patients with T2DM.

At present, very limited types of drugs exist for the treatment of childhood diabetes. Metformin is the only oral therapy for youth with type 2 diabetes, even though up to 50% require additional agents within two years of diagnosis (23). Therefore, the current status of available treatments for children with type 2 diabetes is not optimistic. Insulin is generally indicated, despite being inconvenient to use and prone to causing hypoglycemia. Sulfonylurea sensitivity is a main feature of *ABCC8* MODY (24). However, no studies have reported whether sulfonylureas are safe as a treatment for pediatric diabetes patients in China, especially in children with the MODY12 subtype. In this study, we found that the patients belonged to the MODY12 subtype, and consequently we tried to recommend oral sulfonylureas as medication, although there is no relevant experience or evidence of the use of sulfonylureas in children. Proband A, an eight-year-old female, exhibited high levels of both fasting blood glucose and glycosylated hemoglobin, and was, therefore, prescribed high-dose insulin and oral hypoglycemic agents (insulin glulisine, 20 units, three times daily; degludec, 30 units/night; metformin tablets, 850 mg, twice daily; and pioglitazone hydrochloride and metformin hydrochloride tablets, 15 mg/500mg twice daily) for two days. Nonetheless, her FPG levels did not decrease and we added a small dose of glimepiride, which caused her blood sugar to drop promptly. Thus, the insulin dose was gradually reduced, while the dose of glimepiride was increased to the point where insulin administration was completely ceased and only glimepiride (2 mg/day) was administered. However, proband A developed ketosis, a phenomenon that also occurred in proband B, moreover, the dosage of glimepiride was increased to 4 mg/day in both patients, and the patients’ blood glucose levels returned to the normal range. In summary, individuals with *ABCC8* mutations are sensitive to sulfonylureas. The dosage of sulfonylureas is very important for children with *ABCC8* mutations, and 4mg/day glimepiride was effective in both adults and children. In the cases where fasting blood sugar levels are abnormally high and the use of insulin cannot control blood sugar, it is recommended to add a low dose of sulfonylurea such as glimepiride, which has mild hypoglycemic effect. In addition, sulfonylureas are not currently recommended for juvenile diabetes patients in China. Therefore, China’s guidelines should recommend using sulfonylureas to prevent and treat childhood diabetes in patients with MODY12.

Glucagon-like peptide 1 (GLP-1) is a proglucagon cleavage product produced in intestinal L cells, which is secreted primarily after ingestion of glucose or a mixed meal. It increases glucose-stimulated insulin secretion at physiological plasma concentrations (25). GLP-1 receptor agonists provide the possibility to improve glycemic control and reduce body weight in type 2 diabetes without the risk of hypoglycemia, and its blood sugar reduction effect is significantly better than that of currently used drugs (25, 26).Although GLP-1 receptor agonists are currently used only for the treatment of type 2 diabetes (27), GLP-1 receptor agonists are also used to treat MODY patients, especially MODY4 (28) since this subtype is mainly characterized by type 2 diabetes. Although there is no relevant experience or evidence of usingGLP-1 receptor agonists in children in China, expert guidance on the clinical application of GLP-1 receptor agonists does not recommend using GLP-1 receptor agonists to treat adolescents with diabetes. In the present study, the patients presented with type 2 diabetes as well and were very sensitive to GLP-1 receptor agonists. Therefore, we postulate that GLP-1 receptor agonists are effective hypoglycemic drugs for treating children with type 2 diabetes, consistent with reports abroad (29). Current GLP-1 receptor agonists are mainly administered by subcutaneous injections. However, semaglutide is the first orally administered GLP-1 receptor agonist, which was approved by the U.S. Food and Drug Administration in September 2019 for adults with type 2 diabetes, and has been approved by the European Medicines Agency (30). Oral GLP-1 receptor agonists may exhibit greater tolerability than their subcutaneous counterparts.

In family A, although proband A’s father (currently 38 years old) carried this mutation site (c.C1555T), he did not have diabetes (FPG, 5.12 mmol/L; HbA_1C_, 5.4%; urine glucose, negative). According to reports in the literature (31, 32), we speculate that, first, individuals with *ABCC8* mutations are diagnosed with diabetes across a wide age range (33); second, the onset of diabetes may be modified by certain genes (32, 34), delaying the age of onset in individuals with *ABCC8* mutations or even suppressing diabetes symptoms. In addition, proband A did not respond to 50 units of insulin/day, and her triglyceride levels were 50.21 mmol/L, so she might be in a state of insulin resistance. In family B, proband B’s father may also have a genetic history of diabetes, or the child could possibly be a compound heterozygote with an unidentified mutation in the other chromosome from his mother, which would aggravate the diabetic phenotype. This could also be a coincidence, and the *ABCC8* mutation might play a leading role in the occurrence of diabetes. In addition, patients with MODY12 subtype presented with diabetic retinopathy or neuropathy, which reinforces the need for precise treatment for these patients. This would contribute to controlling blood sugar in a targeted manner, to prevent complications and to improve the quality of life of patients. However, the functional significance of the above three variants in the *ABCC8* gene was unclear (VUS). Although we found that these variants may be pathogenic mutations, their function needs to be further verified and should not be used to guide clinical management. In this study, three mutations (p. R519C, p. I1236V, and p. S962L) were rare according to the public database. However, patients with these mutations are sensitive to sulfonylureas or liraglutide. Therefore, patients with an age of onset of younger than 25 years old, a significant family history of diabetes, and negative test results for diabetes autoantibodies, should undergo diabetes-related genetic screening, and appropriate drugs and dosages should be selected according to the patient’s genotype.

To date, 14 different genes have been reported to cause diabetes with MODY-like phenotypes, and each MODY subtype has different patterns, such as the age at onset, clinical features, and therapeutic strategies (35). MODY accounts for 1-5% of all cases of diabetes (8).With improved knowledge and awareness of monogenic diabetes and the development of more accurate and inexpensive molecular diagnostic techniques, the diagnosis rate of MODY will increase, and the results of genetic diagnosis will provide the most precise treatment plans for the different types of MODY patients.

In conclusion, *ABCC8* gene mutations c.1555 C>T (p.R519C), c.3705 A>G (p.I1236V), and c.2885 C>T (p.S962L) were the respective pathogenic mutations in three *ABCC8*-MODY pedigrees. Glimepiride (2 mg/day) was not effective in controlling blood sugar in children with *ABCC8* mutations. However, 4 mg/day glimepiride was effective in both adults and children. Moreover, liraglutide was effective in controlling blood sugar in patients with *ABCC8* mutations in both adults and children. Precise treatment based on individual genotype not only improves treatment effects and reduces adverse reactions, but also saves limited medical resources.

## Authors contributions

Conceived and designed the experiments, JL, HZ, and YL. Performed the experiments, JL, YL, and AD. Analyzed the data, JL, LW, CL, HM, and XW. Wrote the paper, JL. JL, XW, and HM contributed equally. All authors contributed to the article and approved the submitted version.

## Data availability statement

The datasets for this article are not publicly available due to concerns regarding participant/patient anonymity. Requests to access the datasets should be directed to the corresponding authors.

## Ethics statement

The studies involving human participants were reviewed and approved by ethics committee of the Central Hospital of Wuhan. Written informed consent to participate in this study was provided by the participants’ legal guardian/next of kin. Written informed consent was obtained from the individual(s), and minor(s)’ legal guardian/next of kin, for the publication of any potentially identifiable images or data included in this article.

## Funding

This study was supported by the National Natural Science Foundation of China (No. 81900719, No. 81800704 and No. 81870173) and the Health and Family Planning Commission of Wuhan City (No. WX18M02).

## Acknowledgments

We thank the probands and their families for their support and participation.

## Conflict of interest

The authors declare that the research was conducted in the absence of any commercial or financial relationships that could be construed as a potential conflict of interest.

## Publisher’s note

All claims expressed in this article are solely those of the authors and do not necessarily represent those of their affiliated organizations, or those of the publisher, the editors and the reviewers. Any product that may be evaluated in this article, or claim that may be made by its manufacturer, is not guaranteed or endorsed by the publisher.

## Supplementary material

Supplementary Table 1 Sanger sequencing results of candidate gene loci (*ABCC8*) in the three family members. HGVSc: human genome variation societycDNA; HGVSp: human genome variation society protein; ALT: alternative; REF: reference

Supplementary Table 2 Pathogenicity results of candidate gene mutation sites (*ABCC8*) predicted by bioinformatics. HGVSc:human genome variation societycDNA; SIFT: Deleterious(<0.05); PolyPhen2_HVAR: Probably damaging (>=0.909), possibly damaging (0.447<=pp2_hdiv<=0.909); benign (<=0.446); PolyPhen2_HDIV: Probably damaging (>=0.957), possibly damaging (0.453<=pp2_hdiv<=0.956); benign (<=0.452); MutationTaster: Deleterious (>0.5); LRT: lower scores are more deleterious; MutationAssessor: Deleterious (>1.938); FATHMM: Deleterious (<-1.5); SiPhy_29way, PhyloP46way and PhyloP100way: higher scores are more deleterious; CADD: Deleterious (>15); Gerp++: Deleterious (>2); MCAP: Deleterious (>0.025); REVEL: reference range 0-1.

Supplementary Table 3 Evolutionary conservation analysis for the three mutations in *ABCC8.*NP_001274103.1 match to NM_001287174.3; Proban **(A)** *ABCC8*:NM_001287174:exon10:c.C1555T:p.R519C; Proban **(B)** *ABCC8*:NM_001287174:exon30:c.A3706G:p.I1236V; Proban **(C)** *ABCC8*:NM_001287174:exon24:c.C2885T:p.S962L.
