# Supplementary material for: Precision therapy for three Chinese families with maturity-onset diabetes of the young (MODY12)
Source: Front Endocrinol (Lausanne). 2022 Aug 3;13:858096. doi: 10.3389/fendo.2022.858096 (PMC9381955; doi:10.3389/fendo.2022.858096)
Supplement: Supplementary Table 1 — Sanger sequencing results of candidate gene loci (ABCC8) in the three family members. HGVSc: human genome variation societycDNA; HGVSp: human genome variation society protein; ALT: alternative; REF: reference [file Table_1.docx]

Table **S1:** Sanger sequencing results of candidate gene loci (*ABCC8*) in the three family members

| **Proband** | **HGVSc** | **HGVSp** | **Member of family** | **ALT** | **REF** |
| --- | --- | --- | --- | --- | --- |
| A | c.1555 C>T | p.R519C | Ⅱ-1 | T | C |
|  |  |  | Ⅱ-2 | C | C |
|  |  |  | Ⅲ-1 | T | C |
|  |  |  | Ⅲ-2 | C | C |
|  |  |  | IV-1 | T | C |
|  |  |  |  |  |  |
| B | c.3706 A>G | p.I1236V | Ⅱ-1 | A | A |
|  |  |  | Ⅱ-2 | A | A |
|  |  |  | Ⅱ-3 | A | A |
|  |  |  | Ⅱ-4 | G | A |
|  |  |  | Ⅱ-5 | G | A |
|  |  |  | Ⅱ-6 | G | A |
|  |  |  | Ⅲ-1 | A | A |
|  |  |  | Ⅲ-2 | G | A |
|  |  |  | Ⅲ-3 | A | A |
|  |  |  | IV-1 | G | A |
|  |  |  |  |  |  |
| C | c.2885 C>T | p.S962L | Ⅱ-4 | T | C |
|  |  |  | Ⅲ-1 | T | C |

HGVSc: [human genome variation society](http://www.baidu.com/link?url=OE6YTtSqK677yw6k3uIElpq5WDzqDzLMg6kBOHo9nAO)cDNA; HGVSp: [human genome variation society](http://www.baidu.com/link?url=OE6YTtSqK677yw6k3uIElpq5WDzqDzLMg6kBOHo9nAO) protein; ALT: alternative; REF: reference
