# Supplementary material for: Precision therapy for three Chinese families with maturity-onset diabetes of the young (MODY12)
Source: Front Endocrinol (Lausanne). 2022 Aug 3;13:858096. doi: 10.3389/fendo.2022.858096 (PMC9381955; doi:10.3389/fendo.2022.858096)
Supplement: Supplementary Table 2 — Pathogenicity results of candidate gene mutation sites (ABCC8) predicted by bioinformatics. HGVSc:human genome variation societycDNA; SIFT: Deleterious(<0.05); PolyPhen2_HVAR: Probably damaging (>=0.909), possibly damaging (0.447<=pp2_hdiv<=0.909); benign (<=0.446); PolyPhen2_HDIV: Probably damaging (>=0.957), possibly damaging (0.453<=pp2_hdiv<=0.956); benign (<=0.452); MutationTaster: Deleterious (>0.5); LRT: lower scores are more deleterious; MutationAssessor: Deleterious (>1.938); FATHMM: Deleterious (<-1.5); SiPhy_29way, PhyloP46way and PhyloP100way: higher scores are more deleterious; CADD: Deleterious (>15); Gerp++: Deleterious (>2); MCAP: Deleterious (>0.025); REVEL: reference range 0-1. [file Table_2.docx]

Table **S2:** Pathogenicity results of candidate gene mutation sites (*ABCC8*) predicted by bioinformatics

| **Proband** | **HGVSc** | **SIFT** | **PolyPhen2_HVAR** | **PolyPhen2_HDIV** | **MutationTaster** | **LRT** | **MutationAssessor** | **FATHMM** | **SiPhy­_29way_logOdds** |
| --- | --- | --- | --- | --- | --- | --- | --- | --- | --- |
| A | c.C1555T | 0.976 | 0.0 | 0.0 | 0.886039 | 0.000927 | -0.59 | -2.56 | 9.9279 |
| B | c.A3706G | 0.04 | 0.216 | 0.257 | 0.999993 | 0.000000 | 1.205 | -3.39 | 15.5267 |
| C | c.C2885T | 0.438 | 0.279 | 0.944 | 1 | 0.000000 | 2.48 | -2.98 | 20.8794 |

continuted Table S2

| **Proband** | **HGVSc** | **PhyloP46way_placental** | **PhyloP100way_vertebrate** | **CADD** | **Gerp++gt2** | **MCAP** | **REVEL** |
| --- | --- | --- | --- | --- | --- | --- | --- |
| A | c.C1555T | - | - | - | - | 0.077062356 | 0.215 |
| B | c.A3706G | 2.082 | 5.115 | - | 5.46 | 0.04573512 | 0.119 |
| C | c.C2885T | 2.941 | 7.104 | 4.342716 | 6.17 | 0.051035 | 0.482 |

HGVSc：[human genome variation society](http://www.baidu.com/link?url=OE6YTtSqK677yw6k3uIElpq5WDzqDzLMg6kBOHo9nAO)cDNA; SIFT: Deleterious(<0.05); PolyPhen2_HVAR: Probably damaging (>=0.909), possibly damaging (0.447<=pp2_hdiv<=0.909); benign (<=0.446); PolyPhen2_HDIV: Probably damaging (>=0.957), possibly damaging (0.453<=pp2_hdiv<=0.956); benign (<=0.452); MutationTaster: Deleterious (>0.5); LRT: lower scores are more deleterious; MutationAssessor: Deleterious (>1.938); FATHMM: Deleterious (<-1.5); SiPhy­_29way, PhyloP46way and PhyloP100way: higher scores are more deleterious; CADD: Deleterious (>15); Gerp++: Deleterious (>2); MCAP: Deleterious (>0.025); REVEL: reference range 0-1.
