# Supplementary material for: Precision therapy for three Chinese families with maturity-onset diabetes of the young (MODY12)
Source: Front Endocrinol (Lausanne). 2022 Aug 3;13:858096. doi: 10.3389/fendo.2022.858096 (PMC9381955; doi:10.3389/fendo.2022.858096)
Supplement: Supplementary Table 3 — Evolutionary conservation analysis for the three mutations in ABCC8.NP_001274103.1 match to NM_001287174.3; Proban (A) ABCC8:NM_001287174:exon10:c.C1555T:p.R519C; Proban (B) ABCC8:NM_001287174:exon30:c.A3706G:p.I1236V; Proban (C) ABCC8:NM_001287174:exon24:c.C2885T:p.S962L. [file Table_3.docx]

Table **S3:**Evolutionary conservation analysis for the three mutations in *ABCC8*

| **Protein Acc.** | **Gene** | **Organism** | **Amino acid sequences** | | |
| --- | --- | --- | --- | --- | --- |
| **Proband A** |  |  |  | | |
| [NP_001274103.1](https://www.ncbi.nlm.nih.gov/entrez/query.fcgi?cmd=Retrieve&db=protein&list_uids=562815400&dopt=GenPept&term=562815400&qty=1&linkbar=jsmenu2) | *ABCC8* | H.sapiens | 499 | TNEMLRGIKLLKLYAWENIFRTRVETTRRKEMTSLRAFAIYTSISIFMNT | 548 |
| [XP_508310.4](https://www.ncbi.nlm.nih.gov/entrez/query.fcgi?cmd=Retrieve&db=protein&list_uids=410044939&dopt=GenPept&term=410044939&qty=1&linkbar=jsmenu2) | *ABCC8* | P.troglodytes | 535 | TNEMLRGIKLLKLYAWENIFRTRVETTRRKEMTSLRAFAIYTSISIFMNT | 584 |
| [XP_001088694.1](https://www.ncbi.nlm.nih.gov/entrez/query.fcgi?cmd=Retrieve&db=protein&list_uids=109107151&dopt=GenPept&term=109107151&qty=1&linkbar=jsmenu2) | *ABCC8* | M.mulatta | 499 | TNEMLRGIKLLKLYAWENIFRTRVETTRRKEMTSLRAFAIYTSISIFMNT | 548 |
| [XP_005633781.1](https://www.ncbi.nlm.nih.gov/entrez/query.fcgi?cmd=Retrieve&db=protein&list_uids=545536965&dopt=GenPept&term=545536965&qty=1&linkbar=jsmenu2) | *ABCC8* | C.lupus | 499 | TNEMLRGIKLLKLYAWENIFCTRVEMTRRKEMTSLRAFAVYTSISIFMNT | 548 |
| [NP_001192539.1](https://www.ncbi.nlm.nih.gov/entrez/query.fcgi?cmd=Retrieve&db=protein&list_uids=329663587&dopt=GenPept&term=329663587&qty=1&linkbar=jsmenu2) | *ABCC8* | B.taurus | 499 | TNEMLRGIKLLKLYAWENIFRTRVEMTRKKEMTSLRAFAICTSISIFMNT | 548 |
| [NP_035640.2](https://www.ncbi.nlm.nih.gov/entrez/query.fcgi?cmd=Retrieve&db=protein&list_uids=62241017&dopt=GenPept&term=62241017&qty=1&linkbar=jsmenu2) | *Abcc8* | M.musculus | 499 | TNEMLRGIKLLKLYAWENIFCSRVEMTRRKEMTSLRAFAVYTSISIFMNT | 548 |
| [NP_037171.2](https://www.ncbi.nlm.nih.gov/entrez/query.fcgi?cmd=Retrieve&db=protein&list_uids=148368981&dopt=GenPept&term=148368981&qty=1&linkbar=jsmenu2) | *Abcc8* | R.norvegicus | 499 | TNEMLRGIKLLKLYAWENIFCSRVEKTRRKEMTSLRAFAVYTSISIFMNT | 548 |
| [XP_421005.2](https://www.ncbi.nlm.nih.gov/entrez/query.fcgi?cmd=Retrieve&db=protein&list_uids=118091216&dopt=GenPept&term=118091216&qty=1&linkbar=jsmenu2) | *ABCC8* | G.gallus | 515 | TNEMLRGIKLLKLYAWEHIFHSSVEETRQKEMTSLKSFALYTSISIFMNA | 564 |
| [NP_001166118.2](https://www.ncbi.nlm.nih.gov/entrez/query.fcgi?cmd=Retrieve&db=protein&list_uids=310688879&dopt=GenPept&term=310688879&qty=1&linkbar=jsmenu2) | abcc8 | D.rerio | 499 | TNELLRGIKLLKLYAWEHIFCSNVEETRCKELTSLQAFALYTSISIFMNT | 548 |
|  |  |  |  |  |  |
| **Proband B** |  |  |  |  |  |
| [NP_001274103.1](https://www.ncbi.nlm.nih.gov/entrez/query.fcgi?cmd=Retrieve&db=protein&list_uids=562815400&dopt=GenPept&term=562815400&qty=1&linkbar=jsmenu2) | *ABCC8* | H.sapiens | 1236 | IASLFLTAANRWLEVRMEYIGACVVLIAAVTSISNSLHRELSAGLVGLGL | 1285 |
| [XP_508310.4](https://www.ncbi.nlm.nih.gov/entrez/query.fcgi?cmd=Retrieve&db=protein&list_uids=410044939&dopt=GenPept&term=410044939&qty=1&linkbar=jsmenu2) | *ABCC8* | P.troglodytes | 1272 | IASLFLTAANRWLEVRMEYIGACVVLIAAVTSISNSLHRELSAGLVGLGL | 1321 |
| [XP_001088694.1](https://www.ncbi.nlm.nih.gov/entrez/query.fcgi?cmd=Retrieve&db=protein&list_uids=109107151&dopt=GenPept&term=109107151&qty=1&linkbar=jsmenu2) | *ABCC8* | M.mulatta | 1235 | IASLFLTAANRWLEVRMEYIGACVVLIAAVTSISNSLHRELSAGLVGLGL | 1284 |
| [XP_005633781.1](https://www.ncbi.nlm.nih.gov/entrez/query.fcgi?cmd=Retrieve&db=protein&list_uids=545536965&dopt=GenPept&term=545536965&qty=1&linkbar=jsmenu2) | *ABCC8* | C.lupus | 1235 | IASLFLTAANRWLEVRMEYIGACVVLIAAVTSISNSLHRELSAGLVGLGL | 1284 |
| [NP_001192539.1](https://www.ncbi.nlm.nih.gov/entrez/query.fcgi?cmd=Retrieve&db=protein&list_uids=329663587&dopt=GenPept&term=329663587&qty=1&linkbar=jsmenu2) | *ABCC8* | B.taurus | 1235 | IASLFLTAANRWLEVRMEYIGACVVLIAAVTSISNSLHKELSAGLVGLGL | 1284 |
| [NP_035640.2](https://www.ncbi.nlm.nih.gov/entrez/query.fcgi?cmd=Retrieve&db=protein&list_uids=62241017&dopt=GenPept&term=62241017&qty=1&linkbar=jsmenu2) | *Abcc8* | M.musculus | 1242 | IASLFLTAANRWLEVRMEYIGACVVLIAAATSISNSLHRELSAGLVGLGL | 1291 |
| [NP_037171.2](https://www.ncbi.nlm.nih.gov/entrez/query.fcgi?cmd=Retrieve&db=protein&list_uids=148368981&dopt=GenPept&term=148368981&qty=1&linkbar=jsmenu2) | *Abcc8* | R.norvegicus | 1236 | IASLFLTAANRWLEVRMEYIGACVVLIAAATSISNSLHRELSAGLVGLGL | 1285 |
| [XP_421005.2](https://www.ncbi.nlm.nih.gov/entrez/query.fcgi?cmd=Retrieve&db=protein&list_uids=118091216&dopt=GenPept&term=118091216&qty=1&linkbar=jsmenu2) | *ABCC8* | G.gallus | 1249 | IASLFLTAANRWLEVRMEYIGACVVLIAAVTSITSCLYRNLSSGLVGLGL | 1298 |
| [NP_001166118.2](https://www.ncbi.nlm.nih.gov/entrez/query.fcgi?cmd=Retrieve&db=protein&list_uids=310688879&dopt=GenPept&term=310688879&qty=1&linkbar=jsmenu2) | *abcc8* | D.rerio | 1235 | MASLFLTAANRWLEVRMEYIGACIVLIAAVASITNSLYSHLSTGLVGLGL | 1284 |
|  |  |  |  |  |  |
| **Proband C** |  |  |  |  |  |
| [NP_001274103.1](https://www.ncbi.nlm.nih.gov/entrez/query.fcgi?cmd=Retrieve&db=protein&list_uids=562815400&dopt=GenPept&term=562815400&qty=1&linkbar=jsmenu2) | *ABCC8* | H.sapiens | 939 | LEKETVTERKATEPPQGLSRAMSSRDGLLQDEEEEEEEAAESEEDDNLSS | 988 |
| [XP_508310.4](https://www.ncbi.nlm.nih.gov/entrez/query.fcgi?cmd=Retrieve&db=protein&list_uids=410044939&dopt=GenPept&term=410044939&qty=1&linkbar=jsmenu2) | *ABCC8* | P.troglodytes | 975 | LEKETVTERKATEPPKGLSRAMSSRDGLLQDEEEEEEEAAESEEDDNLSS | 1024 |
| [XP_001088694.1](https://www.ncbi.nlm.nih.gov/entrez/query.fcgi?cmd=Retrieve&db=protein&list_uids=109107151&dopt=GenPept&term=109107151&qty=1&linkbar=jsmenu2) | *ABCC8* | M.mulatta | 938 | LEKETVTERKATEPPQGLSRTMSSRDGLLQDEEEEEEEAAESDEDDNLSS | 987 |
| [XP_005633781.1](https://www.ncbi.nlm.nih.gov/entrez/query.fcgi?cmd=Retrieve&db=protein&list_uids=545536965&dopt=GenPept&term=545536965&qty=1&linkbar=jsmenu2) | *ABCC8* | C.lupus | 938 | LEKETVVERKATEPPQGLPRAMSSRDGLLQDEEEEEEEAAESEEEDNLSS | 987 |
| [NP_001192539.1](https://www.ncbi.nlm.nih.gov/entrez/query.fcgi?cmd=Retrieve&db=protein&list_uids=329663587&dopt=GenPept&term=329663587&qty=1&linkbar=jsmenu2) | *ABCC8* | B.taurus | 938 | LEKETVMERKATEPPQGLPRAMSSRDGLLQDEEEEEEEAAESEEEDNLSS | 987 |
| [NP_035640.2](https://www.ncbi.nlm.nih.gov/entrez/query.fcgi?cmd=Retrieve&db=protein&list_uids=62241017&dopt=GenPept&term=62241017&qty=1&linkbar=jsmenu2) | *Abcc8* | M.musculus | 945 | LEKETVMERKAPEPSQGLPRAMSSRDGLLLDEDEEEEEAAESEEDDNLSS | 994 |
| [NP_037171.2](https://www.ncbi.nlm.nih.gov/entrez/query.fcgi?cmd=Retrieve&db=protein&list_uids=148368981&dopt=GenPept&term=148368981&qty=1&linkbar=jsmenu2) | *Abcc8* | R.norvegicus | 939 | LEKETVMERKAPEPSQGLPRAMSSRDGLLLDEDEEEEEAAESEEDDNLSS | 988 |
| [XP_421005.2](https://www.ncbi.nlm.nih.gov/entrez/query.fcgi?cmd=Retrieve&db=protein&list_uids=118091216&dopt=GenPept&term=118091216&qty=1&linkbar=jsmenu2) | *ABCC8* | G.gallus | 952 | LEKETTIETKTVLERTNLRRPMYSREALLKDDEEDEEEATESDDEDNLSS | 1001 |
| [NP_001166118.2](https://www.ncbi.nlm.nih.gov/entrez/query.fcgi?cmd=Retrieve&db=protein&list_uids=310688879&dopt=GenPept&term=310688879&qty=1&linkbar=jsmenu2) | *abcc8* | D.rerio | 937 | FEKETVDENMTVLERKNLRRAMYSREALKTEEDEEEESLESDDDDNLSQ | 985 |

NP_001274103.1 match to [NM_001287174.3](https://www.ncbi.nlm.nih.gov/nuccore/NM_001287174.3);

Proban A: *ABCC8*:NM_001287174:exon10:c.C1555T:p.R519C;

Proban B: *ABCC8*:NM_001287174:exon30:c.A3706G:p.I1236V;

Proban C: *ABCC8*:NM_001287174:exon24:c.C2885T:p.S962L
